# ASR technology in college English speaking instruction: the role of feedback internalization and metacognitive strategies

**DOI:** 10.3389/fpsyg.2026.1847238

**Published:** 2026-09-11

**Authors:** Dumei Chen, Lining Zeng, Weixing Ou, Shihui Zhong

**Affiliations:** 1 School of Educational Science, Hunan Normal University, Changsha, China; 2 School of Foreign Languages, Changsha Normal University, Changsha, China; 3 Hunan University of Finance and Economics, Changsha, China; 4 Hunan Academy of Education Sciences, Changsha, China; 5 School of Civil Engineering, Changsha University of Science and Technology, Changsha, China

**Keywords:** automatic speech recognition, feedback internalization, language proficiency, learning motivation, metacognitive strategies

## Abstract

This study examines how Automatic Speech Recognition (ASR) technology can enhance college English speaking instruction by analyzing its interaction with metacognitive strategies and learner differences. Using questionnaire surveys, factor analysis, and structural equation modeling, we assess how ASR-generated feedback quality, structured reflection tasks, and usage frequency influence feedback internalization, reflective learning, and motivation. The results show that accurate error correction and well-designed reflection tasks significantly improve feedback processing and reflective behavior, while frequent ASR use primarily boosts reflection rather than motivation. Interestingly, ASR accuracy enhances intrinsic motivation but does not directly drive reflection. Language proficiency emerges as a key moderator, with more proficient learners internalizing feedback more effectively and achieving greater speaking gains. The study contributes to second language acquisition theory by integrating metacognitive strategies with ASR-assisted learning. It provides empirical support for intelligent teaching systems and practical guidance for improving oral English instruction, intercultural communication training, and personalized learning. By bridging technology and pedagogy, this research advances active learning methodologies, enhances speaking proficiency, and strengthens the global competitiveness of higher education.

## 1 Introduction

In today’s globalized world, English serves as the primary medium for international communication, making effective oral proficiency essential for academic and professional success (Kubota, 2022). This demand is reflected in recent reports: approximately 85% of global employers prioritize English communication skills in hiring decisions, while 63% of non-native English-speaking university students actively seek to improve their oral proficiency. In response, 78% of national education systems worldwide have incorporated English speaking enhancement into their core curricula. However, traditional oral English instruction often faces challenges in providing timely feedback and adapting to individual learner needs, failing to address learners’ individual needs or keep pace with global communication requirements (Nakamura and Sakurai, 2024).

The emergence of ASR technology offers a promising avenue for addressing these limitations of delayed feedback and uniform instructional approaches. Unlike conventional methods, ASR technology provides immediate, personalized feedback on pronunciation accuracy, and support for structured reflection tasks that foster metacognitive awareness. This technological advancement enables learners to identify and correct errors in real-time while developing crucial self-regulation skills (Uchihara and Clenton, 2023). However, current research has largely overlooked the complex interplay between technological features and learner-specific factors that ultimately determine the effectiveness of ASR-assisted instruction. Specifically, little attention has been paid to how individual differences in language proficiency and learning motivation interact with metacognitive strategies to shape the feedback internalization process (Molenaar et al., 2023).

To address this gap, the present study develops a comprehensive theoretical framework that examines the dynamic relationships between ASR technology, learner characteristics, and pedagogical outcomes. At the technological level, we investigate four key ASR features - recognition accuracy, usage frequency, corrective feedback quality, and reflection task design - and their collective impact on learning processes (Ma et al., 2024). The model further explores how these technological features interact with two crucial psychological mediators: reflective behavior, which facilitates the transformation of external feedback into internalized learning, and intrinsic motivation, which sustains engagement with the learning process (Velez et al., 2023). Importantly, the framework accounts for the moderating role of learners’ existing language proficiency, acknowledging that students at different proficiency levels may benefit differently from identical technological interventions.

Through rigorous empirical investigation employing structural equation modeling, this research provides novel insights into the mechanisms through which ASR technology can optimize oral English acquisition. The findings significantly advance theoretical understanding in three key areas: the application of self-regulated learning principles in technology-enhanced environments, the role of metacognition in feedback processing, and the importance of adaptive design in educational technology. Practically, the study offers practical guidance for educators seeking to implement ASR technology effectively, for developers aiming to create more responsive learning tools, and for policymakers working to align language education with 21st-century communication needs. By demonstrating how ASR technology can be tailored to diverse learner profiles and integrated with sound pedagogical principles, this research supports the development of more equitable and effective oral English instruction for an increasingly interconnected world.

## 2 Literature review

While much research has focused on ASR technology’s capacity to measure oral proficiency, the immediate concern of this study is the instructional process, that is, how ASR can be harnessed to design more effective speaking pedagogy. This pedagogical focus constitutes the study’s core novelty.

### 2.1 ASR technology in language learning

ASR technology has emerged as a transformative tool in language education, offering real-time, personalized feedback on learners’ oral production. Unlike traditional instruction, where feedback is often delayed and generic, ASR technology provides immediate error correction that enables learners to identify and address pronunciation, intonation, and grammatical issues during practice (Golonka et al., 2014; Thomson and Derwing, 2015). This immediate feedback mechanism not only enhances learners’ awareness of target language features but also fosters the development of self-regulated learning skills (Benson, 2011).

The effectiveness of the ASR system in oral instruction depends on four core features. First, recognition accuracy refers to the system’s ability to accurately capture and identify learners’ speech errors. High recognition accuracy helps learners pinpoint problematic areas and provides a reliable basis for subsequent improvement (McCrocklin, 2016; Liakin et al., 2017). Second, usage frequency reflects the density of learners’ interaction with the system. Frequent use not only provides ample practice opportunities but also reinforces learning through continuous immediate feedback (Neri et al., 2008). Third, corrective feedback refers to the immediate suggestions provided by the system for errors in pronunciation, intonation, and grammar. Research indicates that clear and timely corrective feedback can stimulate learners’ deep reflection and transform external feedback into concrete improvement strategies (Li, 2010). Fourth, reflection tasks include structured activities such as listening to recordings, comparing correct and incorrect expressions, and writing reflective journals. These tasks help learners internalize external feedback into improvement measures and provide a framework for self-assessment that gradually develops self-regulation (van Lier, 2007; Oxford, 2017). These four features collectively provide data support and a cognitive framework for feedback internalization and knowledge transfer (Chapelle, 2003; Levy and Stockwell, 2006). ASR technology not only helps learners quickly identify and correct language deviations but also promotes the use of metacognitive strategies through guided reflection tasks.

Despite these benefits, researchers have noted potential psychological drawbacks of ASR use. Several studies have shown that while ASR can improve pronunciation accuracy, it may also lead to decreased self-confidence as learners realize that their actual pronunciation falls short of their prior self-perceptions (Saito et al., 2020; Suzuki, 2025; Leis, 2025). This phenomenon aligns with the Dunning-Kruger effect, whereby individuals with lower ability overestimate their competence until objective feedback reveals the gap. In the context of ASR-mediated learning, the system’s precise and unfiltered feedback can temporarily discourage learners, particularly those with weaker proficiency who receive frequent error notifications. Consequently, instructional design must balance accurate feedback with supportive scaffolding to maintain learners’ motivation and self-efficacy. How these technological features interact with learners’ individual differences to influence learning outcomes remains an important area for further investigation (Dörnyei, 2005; Plonsky, 2011).

### 2.2 Metacognitive strategies in feedback processing

Metacognitive strategies play a crucial role in transforming external feedback into internalized learning. Defined as the deliberate planning, monitoring, and evaluation of one’s own cognitive processes, metacognitive strategies enable learners to take control of their learning and respond adaptively to feedback (Flavell, 1979; Pintrich, 2000). As Haukas (2018) argues, metacognitive awareness is particularly critical in technology-mediated language learning environments, where learners must navigate autonomous practice without constant teacher guidance. In technology-enhanced language learning environments, these strategies are particularly important because they mediate between technological input and learning outcomes (Vandergrift and Goh, 2012; Zhang and Zhang, 2019).

In ASR-assisted speaking instruction, metacognitive strategies are primarily manifested through two interrelated mechanisms: reflective behavior and engagement with reflection tasks. Reflective behavior refers to the process in which learners review and evaluate their oral expressions, identify gaps between current and target performance, and develop improvement plans (Dewey, 1933; Boud et al., 2013). This reflective process is essential for converting immediate corrective feedback from ASR technology into lasting phonological and grammatical adjustments (Coumel et al., 2023). Empirical research has shown that learners who engage in regular self-reflection after ASR practice demonstrate greater gains in pronunciation accuracy and fluency compared to those who simply repeat exercises without reflection (Chen et al., 2021).

Reflection tasks, as structured teaching activities, provide a framework that scaffolds learners’ metacognitive engagement. These tasks may include listening to recorded speech, comparing one’s production with native speaker models, maintaining reflective journals, and setting specific pronunciation goals (Farrell, 2018; Richards and Lockhart, 1994). Well-designed reflection tasks guide learners through the cyclical process of self-observation, self-judgment, and self-reaction, which are core components of self-regulated learning (Zimmerman, 2000; Schunk and Greene, 2018). Research in computer-assisted language learning indicates that structured reflection tasks significantly enhance learners’ ability to internalize corrective feedback and transfer improved skills to novel speaking contexts (Hauck, 2005; Lai, 2015).

The effectiveness of both reflective behavior and reflection tasks is influenced by learners’ existing metacognitive awareness. Learners with higher metacognitive awareness are better able to recognize the value of feedback, allocate attention to critical features, and adjust their learning strategies accordingly (Wenden, 1998; Oxford, 2017). Conversely, learners with lower metacognitive awareness may benefit from explicit training in how to reflect on ASR feedback and how to use reflection tasks productively (Goh and Burns, 2012; Sato and Loewen, 2022).

Despite the recognized importance of metacognitive strategies in language learning, limited empirical research has examined how these strategies operate specifically within ASR-mediated learning environments. Little is known about how the unique features of ASR technology, such as immediate corrective feedback and automatic reflective prompts, interact with learners’ metacognitive processes, thereby affecting feedback internalization and oral ability development (Plonsky, 2011). The present study addresses this gap by modeling the relationships between ASR features, metacognitive strategies, and learning outcomes.

### 2.3 Language proficiency as a moderator

Individual differences play a fundamental role in second language acquisition, shaping how learners interact with instructional interventions and technological tools (Dörnyei, 2005; Ellis, 2008). Among various individual differences, learners’ existing language abilities, including pronunciation, vocabulary, grammar, and discourse knowledge, have been identified as key moderating factors for feedback effectiveness and learning outcomes in technology mediated environments (DeKeyser, 2012; Li, 2010).

Language proficiency influences learners’ capacity to perceive, process, and internalize external feedback. Students with stronger language proficiency possess more developed phonological categories, larger lexical repertoires, and more automatized grammatical processing, enabling them to quickly and accurately interpret corrective feedback from ASR technology (Kormos, 2014; Segalowitz, 2010). These learners can distinguish between correct and incorrect elements in their output, identify specific areas requiring improvement, and develop effective remediation strategies (Lyster et al., 2013; Trofimovich et al., 2020). Thus, they are better positioned to benefit from the immediate feedback that ASR technology provides.

In contrast, learners with weaker language proficiency may face multiple challenges in feedback processing. Limited phonological perception may prevent them from accurately perceiving the difference between their production and target models (Best and Tyler, 2007; Flege, 1995). Insufficient vocabulary knowledge may impede comprehension of feedback messages, particularly when feedback is delivered textually (Nation, 2013). Underdeveloped grammatical sensitivity may make it difficult to recognize and correct syntactic errors (Ellis, 2006). These learners may require more explicit guidance, repeated exposure, and additional scaffolding to transform ASR feedback into lasting improvement (Goo et al., 2015; Lyster et al., 2013).

In addition to its direct impact on feedback processing, language proficiency also interacts with learners’ emotional responses to ASR technology. Higher proficiency is often associated with greater confidence and lower anxiety when using technology for speaking practice, which in turn enhances engagement and motivation (Gregersen and MacIntyre, 2014; Horwitz et al., 2010). Learners with stronger language proficiency may approach ASR feedback as a useful tool for refinement, whereas less proficient learners may experience frustration or discouragement when feedback highlights multiple errors (Price, 1991; Young, 1991).

The moderating role of language proficiency has been documented across various technology-enhanced learning contexts, such as intelligent tutoring systems for writing (Ranalli, 2021), computer-assisted pronunciation training (McCrocklin, 2016), and web-based collaborative learning environments (Stevenson and Phakiti, 2014). In computer-assisted pronunciation training, studies have found that learners with higher baseline proficiency demonstrate greater gains from ASR-based feedback compared to their less proficient peers (McCrocklin, 2016; Neri et al., 2008). Similar patterns have been observed in intelligent tutoring systems for grammar and writing, where prior knowledge significantly influences the effectiveness of automated feedback (Ranalli, 2021; Stevenson and Phakiti, 2014). These findings suggest that language proficiency may moderate not only the direct effects of feedback but also the mediating pathways through reflective behavior and motivation.

Despite this evidence, limited studies have systematically examined how language proficiency regulates the relationship between ASR technical features, metacognitive strategies, and oral proficiency improvement. It remains unclear whether the effects of corrective feedback and reflection tasks on reflective behavior and motivation are equally strong across proficiency levels, or whether these effects are amplified for more proficient learners (Plonsky, 2011). Addressing this gap, the present study investigates language proficiency as a moderator of the relationships between (a) reflective behavior and oral proficiency improvement, and (b) learning motivation and oral proficiency improvement. Understanding these moderating effects is essential for designing adaptive ASR-based instruction that meets the needs of diverse learners.

## 3 Research model and hypotheses

### 3.1 Conceptual model

**Figure 1:**
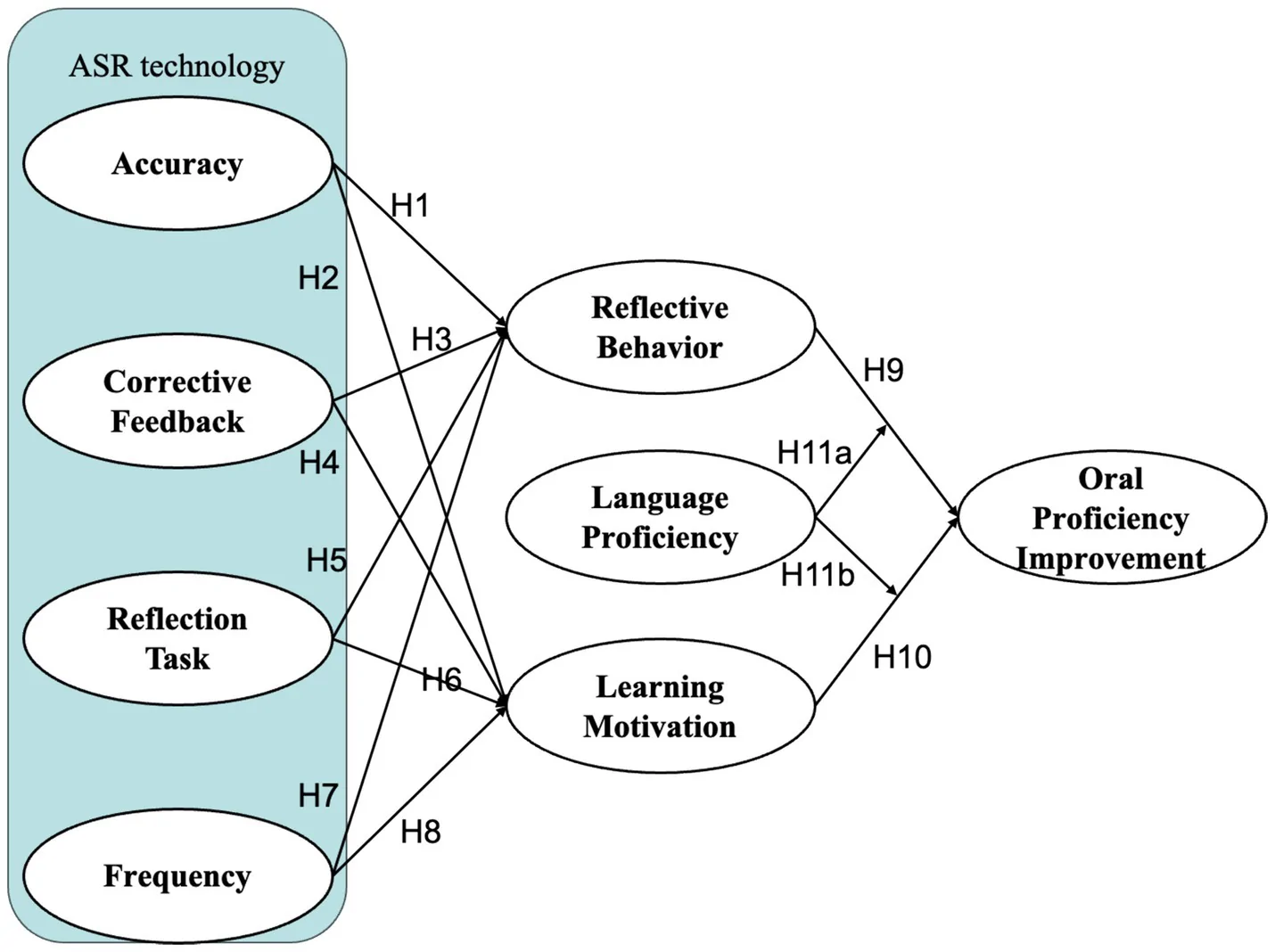
Research model.

Based on the theoretical framework, this study proposes a conceptual model that integrates ASR technological features, metacognitive strategies, and oral proficiency outcomes, with language proficiency as a moderator. The conceptual model is shown as Figure 1.

### 3.2 Effects of ASR features on metacognitive strategies

Accuracy refers to the precision with which the ASR system identifies and diagnoses learners’ speech errors. High recognition accuracy enables learners to pinpoint specific problematic areas in their pronunciation, intonation, and grammar, providing a reliable foundation for subsequent improvement (Inceoglu et al., 2024; McCrocklin, 2016). While accuracy may enhance learners’ focus on errors, its primary effect may be on motivation by building confidence in the feedback received (Liakin et al., 2017). Thus, the following hypotheses are constructed.

*H1*: Accuracy is positively related to reflective behavior.

*H2*: Accuracy is positively related to learning motivation.

Corrective Feedback includes immediate suggestions provided by the ASR system for errors in oral production. Timely and clear corrective feedback helps learners quickly identify shortcomings and encourages proactive reflection and strategy adjustment (Liakina and Liakin, 2023). Research indicates that such feedback can stimulate deep reflection and enhance intrinsic motivation by demonstrating clear pathways for improvement (Li, 2010). Thus, the hypotheses are formulated as follows.

*H3*: Corrective Feedback is positively related to reflective behavior.

*H4*: Corrective Feedback is positively related to learning motivation.

Reflection Tasks include structured activities such as listening to recordings, comparing correct and incorrect expressions, and maintaining reflective journals. These tasks scaffold learners’ metacognitive engagement by providing systematic frameworks for self-assessment (Farrell, 2018; Richards and Lockhart, 1994). Well-designed reflection tasks guide learners through self-observation, self-judgment, and self-reaction, thereby enhancing both reflective behavior and sustained motivation (Zimmerman, 2000; Oxford, 2017). Hence, the following hypotheses are proposed.

*H5*: Reflection Task is positively related to reflective behavior.

*H6*: Reflection Task is positively related to learning motivation.

Frequency reflects the density of learners’ interaction with the ASR system. Frequent use provides abundant practice opportunities and repeated exposure to corrective feedback, which may reinforce learning and encourage habitual reflection (Neri et al., 2008; Golonka et al., 2014). However, the effect of frequency on motivation may be indirect, operating primarily through the accumulation of successful practice experiences. Therefore, the following hypotheses are constructed.

*H7*: Frequency is positively related to reflective behavior.

*H8*: Frequency is positively related to learning motivation.

### 3.3 Effects of metacognitive strategies on oral proficiency improvement

Metacognitive strategies serve as the critical bridge between technological intervention and learning outcomes. Reflective behavior refers to learners’ systematic review and evaluation of their oral expressions, identification of performance gaps, and development of improvement plans (Boud et al., 2013; Dewey, 1933). This reflective process is essential for converting immediate corrective feedback from ASR technology into lasting phonological and grammatical adjustments (Coumel et al., 2023; Vandergrift and Goh, 2012). Learning motivation reflects learners’ intrinsic drive and willingness to invest sustained effort when facing language challenges (Dörnyei, 2005; Gregersen and MacIntyre, 2014). Motivated learners remain positive in the face of setbacks and actively seek improvement through continuous practice and feedback engagement (Horwitz et al., 2010; Dörnyei and Ushioda, 2011). These metacognitive strategies enable learners to transform external feedback into internal learning resources, directly driving oral proficiency improvement. High levels of reflective behavior significantly enhance the recognition and correction of oral errors, while sustained learning motivation provides the necessary energy and emotional support for this process (Plonsky, 2011; Zhang and Zhang, 2019). Thus:

*H9*: Reflective Behavior is positively related to oral proficiency improvement.

*H10*: Learning Motivation is positively related to oral proficiency improvement.

### 3.4 Moderating role of language proficiency

Language Proficiency, learners’ existing abilities in pronunciation, vocabulary, and grammar influences how effectively they can perceive, process, and internalize external feedback (DeKeyser, 2012; Ellis, 2008). Students with stronger language proficiency possess more developed phonological categories, larger lexical repertoires, and more automatized grammatical processing, enabling them to quickly and accurately interpret corrective feedback from ASR technology (Segalowitz, 2010; Kormos, 2014). These learners can distinguish between correct and incorrect elements in their output and develop effective remediation strategies (Trofimovich et al., 2020; Lyster et al., 2013).

In contrast, learners with weaker language proficiency may face challenges in perceiving the difference between their production and target models, comprehending feedback messages, and recognizing syntactic errors (Flege, 1995; Best and Tyler, 2007; Nation, 2013). These learners may require more explicit guidance and repeated exposure to transform ASR feedback into lasting improvement (Goo et al., 2015; Ranalli, 2021). Furthermore, higher proficiency is often associated with greater confidence and lower anxiety when using technology for speaking practice, which enhances engagement and motivation (Horwitz et al., 2010; Price, 1991; Young, 1991). Therefore, language proficiency is hypothesized to moderate the relationships between metacognitive strategies and oral proficiency outcomes.

*H11a*: Language Proficiency moderates the relationship between reflective behavior and oral proficiency improvement.

*H11b*: Language Proficiency moderates the relationship between learning motivation and oral proficiency improvement.

## 4 Methods

### 4.1 Research design

This study’s questionnaire aims to investigate the application effects and influencing mechanisms of ASR technology in college English oral instruction. Its design is strictly based on authoritative literature and empirical findings and adapts measurement items from prior research (see Table 1). To ensure the validity of the measurement instrument, a multi-step validation process was conducted. First, an initial pool of 45 items was generated based on established scales from the literature. Second, five experts in applied linguistics and educational technology reviewed the items for content validity, assessing whether each item adequately represented its intended construct. Based on their feedback, 11 items were revised or removed. Third, a pilot study was conducted with 50 undergraduate students (not included in the main sample) to assess face validity and item clarity. Items with low clarity ratings were reworded accordingly. Fourth, exploratory factor analysis (EFA) and confirmatory factor analysis (CFA) were employed to assess construct validity, as reported in Sections 4.3 and 4.4.

**Table 1:** Sources of questionnaire items.

Variables	Sources
Language proficiency	Marian et al. (2007), MacIntyre et al. (1997)
Learning motivation	Dörnyei (2005), Gregersen and MacIntyre (2014)
Reflective behavior	Boud et al. (2013), Vandergrift and Goh (2012)
Corrective feedback	Li (2010), Lyster et al. (2013)
Reflection task	Farrell (2018), Richards and Lockhart (1994)
Oral proficiency improvement	Council of Europe (2001), Wang and Sun (2020)
Accuracy	Inceoglu et al. (2024), McCrocklin (2016)
Frequency	Golonka et al. (2014), Neri et al. (2008)

The questionnaire is divided into three sections. The first section covers students’ individual characteristics, including Language Proficiency and Learning Motivation. The second section focuses on ASR technology intervention indicators, which include Accuracy, frequency, corrective feedback, and reflection task. The third section addresses feedback internalization and outcome indicators, involving reflective behavior and oral proficiency improvement. All items were rated on a 5-point Likert scale ranging from 1 (strongly disagree) to 5 (strongly agree). The items were adapted from established scales and adjusted to fit the study’s context to ensure comprehensive coverage of theoretical constructs while maintaining strong reliability and validity.

For language proficiency classification, participants were categorized into three levels based on their self-reported common european framework of reference (CEFR) levels and their College English Test (CET) band scores. “Beginner” corresponded to CEFR levels A1-A2 (basic user) and below CET-4 passing score. “Intermediate” corresponded to CEFR levels B1-B2 (independent user) and passing CET-4. “Advanced” corresponded to CEFR levels C1-C2 (proficient user) and passing CET-6. This classification provides readers with a tangible reference for understanding the proficiency distribution of the sample.

This study was approved by the Ethics Committee of Changsha Normal University. All procedures performed in this study involving human participants were in accordance with the ethical standards of the institutional research committee and with the 1964 Helsinki Declaration and its later amendments. The research participants were students recruited from Changsha Normal University. All participants were informed about the purpose of the study, the procedures involved, the potential benefits and risks, and their right to withdraw at any time without penalty. Written informed consent was obtained from all individual participants included in the study. To ensure confidentiality and privacy protection, all data were anonymized prior to analysis, and no identifying information was included in the dataset. Participation in the study was voluntary. Completing the questionnaire took approximately 5–10 min, and no adverse effects were reported or anticipated.

### 4.2 Population and procedure

The participants were undergraduate students recruited from Changsha Normal University, a teacher-training university with multiple disciplines in Hunan Province, China. A total of 400 questionnaires were distributed, with 200 administered online and 200 offline. After screening and data cleaning, 325 valid responses were obtained, yielding a valid response rate of 81.25%. Data were collected from March 1, 2025, to May 20, 2025. The sample included students from various grade levels and academic majors, representing diverse language proficiency levels (beginner: 56%, intermediate: 29%, advanced: 15%).

**Figure 2:**
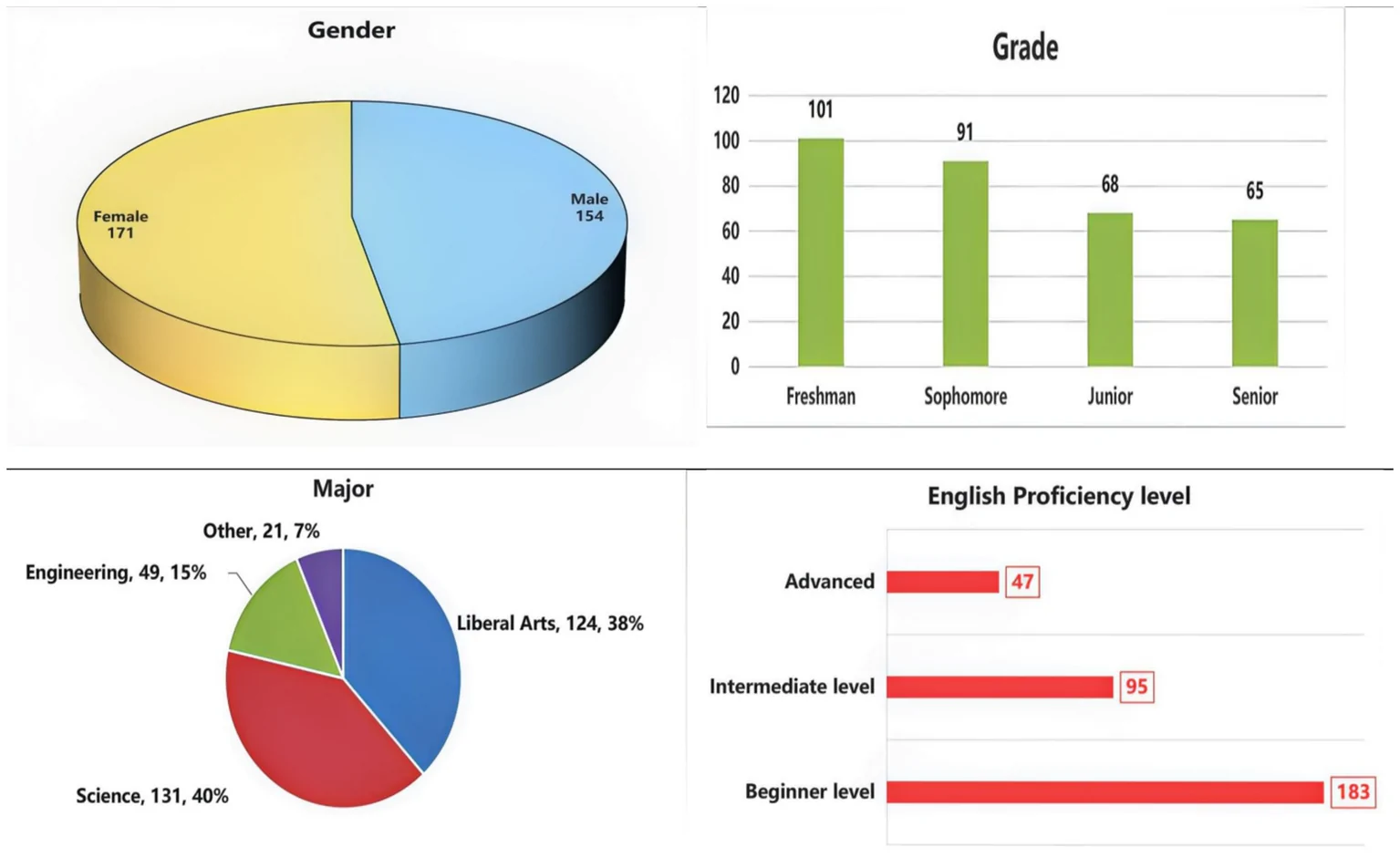
Distribution results of basic information from the questionnaire.

Detailed demographic characteristics of the sample are presented in Figure 2. By collecting data from multiple sources and conducting comprehensive analyses, the questionnaire seeks to fully reveal the role of ASR technology in oral instruction and its impact on students’ language proficiency. This research provides a scientific basis and practical guidance for improving technology-based oral instruction models in higher education. To fully understand the sample composition and validate the scientific rigor and representativeness of subsequent analyses, we performed statistical and visual analyses on basic questionnaire information, including gender, grade level, major, and English proficiency (see Figure 2). Regarding gender distribution, the sample included 154 male participants (47%) and 171 female participants (53%), showing a relatively balanced ratio. While this gender distribution is presented for sample description purposes, subsequent analyses confirmed that gender did not significantly affect the main structural paths, nor did it act as a significant moderator. Therefore, gender was not included as a variable in the main structural equation model.

In terms of grade level, first-year undergraduates numbered 101, followed by second-year students (91), third-year students (68), and fourth-year students (65), showing a decreasing trend by grade. This trend may be due to higher curiosity about new technologies among lower-year students and lower participation among upper-year students because of course, internship, or job search pressures. The major distribution shows that the sample includes 124 humanities students (38%), 131 science students (40%), 49 engineering students (15%), and 21 students from other majors (7%). The high proportion of humanities and science students provides a basis for comparing differences in ASR technology application across academic disciplines. English proficiency is classified into beginner (183, 56%), intermediate (95, 29%), and advanced (47, 15%), based on the CEFR-aligned criteria described in Section 4.1. Most students are at the beginner or intermediate level, indicating a significant potential demand for ASR technology in college English oral instruction. Overall, the sample exhibits diversity in gender, grade level, major, and language proficiency, providing a representative and broadly applicable foundation for subsequent empirical analyses.

### 4.3 Exploratory factor analysis

#### 4.3.1 Reliability and validity analysis

This study aims to investigate the application effects of ASR technology in college English oral instruction. We systematically evaluated the reliability and validity of the questionnaire instrument and employed exploratory factor analysis to test the reasonableness and stability of the data. Table 2 shows that the questionnaire contains 34 items covering eight variables. The Cronbach’s Alpha values for these variables range from 0.783 to 0.895, with an overall Alpha of 0.915. These values indicate high internal consistency and reliability of the measurement constructs. It should be noted that Cronbach’s alpha values exceeding 0.900, as observed for the overall scale, may suggest some degree of item redundancy, indicating that certain items may be measuring very similar aspects of the same construct. While this does not invalidate the reliability of the instrument, future research may consider refining the scale by removing potentially redundant items to improve efficiency without sacrificing reliability. The KMO values for the variables range from 0.789 to 0.887, with an overall KMO of 0.900, far exceeding the standard requirement of 0.7. This demonstrates that the data structure is suitable for factor analysis and has good construct validity. The exploratory factor analysis not only verified the theoretical structure and categorization of the variables but also provided scientific statistical evidence for explaining the intrinsic relationships among the core variables in ASR-assisted oral instruction.

**Table 2:** Reliability and validity.

Variables	Items	Alpha	KMO
Language proficiency	4	0.783	0.789
Learning motivation	4	0.834	0.815
Reflective behavior	4	0.849	0.821
Corrective feedback	4	0.859	0.828
Reflection task	4	0.837	0.813
Oral proficiency improvement	4	0.831	0.814
Accuracy	5	0.852	0.866
Frequency	5	0.895	0.887
Total	34	0.915	0.900

#### 4.3.2 Factor number analysis

This study evaluated whether the questionnaire’s construct dimensions align with theoretical expectations by using exploratory factor analysis and testing the number of factors. The initial eigenvalue analysis (see Table 3) shows that among the 34 components, the first component has an eigenvalue of 9.005 and accounts for 26.485% of the total variance. The eigenvalues for the second through eighth components are 2.794, 2.514, 2.354, 1.937, 1.778, 1.436, and 1.153, respectively. Their individual variance contributions are 8.217, 7.395, 6.924, 5.698, 5.229, 4.224, and 3.392%, with a cumulative contribution of 67.564%. After rotation, the sum of squared loadings for the eight factors are 10.576, 9.483, 8.399, 8.218, 8.039, 7.879, 7.590, and 7.381%. Together, these factors explain 67.564% of the total variance. All initial eigenvalues exceed 1, satisfying Kaiser’s criterion. The rotated factor loadings are well concentrated, revealing a clear factor structure. These results further validate that the eight variables in the questionnaire are both independent and interrelated within the theoretical model.

**Table 3:** Results of factor number analysis.

Item	Initial eigenvalues			Rotated loadings sum of squares		
	Eigenvalue	Variance (%)	Cumulative (%)	Eigenvalue	Variance (%)	Cumulative (%)
1	9.005	26.485	26.485	3.596	10.576	10.576
2	2.794	8.217	34.701	3.224	9.483	20.060
3	2.514	7.395	42.096	2.856	8.399	28.459
4	2.354	6.924	49.021	2.794	8.218	36.676
5	1.937	5.698	54.719	2.733	8.039	44.715
6	1.778	5.229	59.948	2.679	7.879	52.593
7	1.436	4.224	64.172	2.580	7.590	60.183
8	1.153	3.392	67.564	2.510	7.381	67.564

### 4.4 Confirmatory factor analysis

#### 4.4.1 Model index analysis

After conducting exploratory factor analysis, this study further tested the fit of the theoretical model to verify its reasonableness and the stability of the structural relationships among variables. Table 4 shows that the model’s CMIN is 483.199 with 499 degrees of freedom, resulting in a CMIN/DF of 0.968, which is well below the standard threshold of 3. This indicates an excellent overall fit. All fit indices meet or exceed the required standards. For instance, the NFI is 0.911, RFI is 0.900, IFI is 1.003, TLI is 1.004, CFI is 1.000, and GFI is 0.921, all above 0.9. The RMSEA is only 0.001, which is far below the standard of 0.08, further demonstrating minimal model residuals and a high goodness-of-fit. These results show that the theoretical model performs ideally in confirmatory factor analysis, and the relationships among paths and latent variables are strongly supported by the empirical data.

**Table 4:** Model fit indices for confirmatory factor analysis.

Model fit	CMIN	DF	CMIN/DF	NFI	RFI	IFI	TLI	CFI	GFI	RMSEA
Fit results	483.199	499	0.968	0.911	0.900	1.003	1.004	1.000	0.921	0.001
Judgment Std.	–	–	<3	>0.9	>0.9	>0.9	>0.9	>0.9	>0.9	<0.08

#### 4.4.2 Convergent validity and discriminant validity analysis

This study verified the appropriateness and empirical support of each construct measurement tool in the theoretical model through tests of convergent and discriminant validity, ensuring the applicability and scientific rigor of the instruments used. Table 5 shows that all item loadings for each construct are statistically significant. For example, the item loadings for Accuracy are 0.731, 0.747, 0.761, 0.699, and 0.725. The composite reliability (CR) for Accuracy is 0.853, and the average variance extracted (AVE) is 0.537. For Corrective Feedback, item loadings range from 0.751 to 0.819, with a CR of 0.859 and an AVE of 0.603. For Reflection Task, item loadings range from 0.693 to 0.778, with a CR of 0.837 and an AVE of 0.563. For Frequency, item loadings range from 0.769 to 0.820, with a CR of 0.896 and an AVE of 0.631. For Reflective Behavior, the item loadings range from 0.727 to 0.791, with a CR of 0.850 and an AVE of 0.585. For Learning Motivation, item loadings range from 0.708 to 0.774, with a CR of 0.834 and an AVE of 0.557. For Oral Proficiency Improvement, the item loadings are 0.719, 0.754, 0.742, and 0.751, with a CR of 0.830 and an AVE of 0.550. For Language Proficiency, the item loadings range from 0.659 to 0.712, with a CR of 0.783 and an AVE of 0.474. Although the AVE for Language Proficiency is slightly below the ideal level, all constructs meet the convergent validity standard. This indicates that the measurement indicators effectively explain the variance of their corresponding constructs and exhibit high internal consistency. Table 6 shows that the correlation coefficients among constructs are generally low and are lower than the square root of the AVE for each construct (for example, the square root of accuracy is approximately 0.733). This demonstrates that there are no significant cross-loadings among constructs and that they are clearly distinct. Overall, the results of the convergent and discriminant validity tests are satisfactory. Each construct not only fully reflects its theoretical connotation but also shows clear independence and distinction from other constructs.

**Table 5:** Convergent validity and composite reliability.

Construct	Item	Loading factor	CR	AVE
Accuracy	AC5	0.731	0.853	0.537
	AC4	0.747		
	AC3	0.761		
	AC2	0.699		
	AC1	0.725		
Corrective feedback	CF4	0.751	0.859	0.603
	CF3	0.764		
	CF2	0.819		
	CF1	0.771		
Reflection task	RT4	0.778	0.837	0.563
	RT3	0.744		
	RT2	0.782		
	RT1	0.693		
Frequency	FR5	0.796	0.896	0.631
	FR4	0.815		
	FR3	0.772		
	FR2	0.769		
	FR1	0.82		
Reflective behavior	RB1	0.791	0.85	0.585
	RB2	0.769		
	RB3	0.773		
	RB4	0.727		
Learning motivation	LM4	0.774	0.834	0.557
	LM3	0.725		
	LM2	0.777		
	LM1	0.708		
Oral proficiency improvement	OE1	0.719	0.83	0.55
	OE2	0.754		
	OE3	0.742		
	OE4	0.751		
Language proficiency	LF1	0.682	0.783	0.474
	LF2	0.659		
	LF3	0.712		
	LF4	0.701		

**Table 6:** Discriminant validity.

Construct	AC	CF	RT	FR	RB	LM	OE	LF
Accuracy	0.733							
Corrective feedback	0.322	0.777						
Reflection task	0.416	0.291	0.75					
Frequency	0.328	0.209	0.409	0.794				
Reflective behavior	0.264	0.465	0.47	0.332	0.765			
Learning motivation	0.394	0.34	0.368	0.248	0.284	0.746		
Oral proficiency improvement	0.462	0.351	0.455	0.39	0.484	0.319	0.742	
Language proficiency	0.398	0.186	0.365	0.355	0.482	0.221	0.637	0.688

### 4.5 Path analysis of structural equation model

This study used structural equation modeling to test the direct effects among variables. According to the results in Table 7, when examining the relationship between reflective behavior and ASR technology indicators, the path coefficient for recognition accuracy was −0.011 (SE = 0.077, CR = –0.144, *p* = 0.885). This indicates that recognition accuracy has no significant effect on triggering Reflective Behavior. In contrast, timely Corrective Feedback had a significant positive effect on reflective behavior, with a path coefficient of 0.398 (SE = 0.074, CR = 5.416). This finding suggests that the feedback mechanism plays an important role in promoting students’ self-examination and strategy adjustment. Moreover, Reflection Task also had a positive effect on reflective behavior, with a path coefficient of 0.330 (SE = 0.071, CR = 4.620), which is highly statistically significant. The path coefficient for system usage Frequency was 0.131 (SE = 0.057, CR = 2.303, *p* = 0.021), indicating that frequent system use can moderately stimulate reflective behavior. Regarding learning motivation, recognition Accuracy showed a positive effect with a path coefficient of 0.271 (SE = 0.080, CR = 3.380), meaning that higher recognition accuracy helps to enhance learners’ intrinsic drive.

**Table 7:** Path analysis results for the structural equation model.

Hyp.	Path			Estimate	S.E.	C.R.	*p*	Label
H1	Reflective behavior	←	Accuracy	−0.011	0.077	−0.144	0.885	Not supported
H3	Reflective behavior	←	Corrective feedback	0.398	0.074	5.416	< 0.001	Supported
H5	Reflective behavior	←	Reflection task	0.330	0.071	4.620	< 0.001	Supported
H7	Reflective behavior	←	Frequency	0.131	0.057	2.303	0.021	Supported
H2	Learning motivation	←	Accuracy	0.271	0.080	3.380	< 0.001	Supported
H4	Learning motivation	←	Corrective feedback	0.214	0.071	3.008	0.003	Supported
H6	Learning motivation	←	Reflection task	0.185	0.070	2.636	0.008	Supported
H8	Learning motivation	←	Frequency	0.048	0.057	0.837	0.402	Not supported
H10	Oral proficiency improvement	←	Learning motivation	0.210	0.061	3.458	< 0.001	Supported
H9	Oral proficiency improvement	←	Reflective behavior	0.400	0.062	6.479	< 0.001	Supported

Both timely corrective feedback and reflection task also had positive effects on learning motivation, with path coefficients of 0.214 (SE = 0.071, CR = 3.008) and 0.185 (SE = 0.070, CR = 2.636), respectively. However, system usage frequency did not have a significant effect on learning motivation, with a path coefficient of only 0.048 (SE = 0.057, CR = 0.837, *p* = 0.402). In terms of oral proficiency improvement, both intrinsic learning motivation and reflective behavior had direct positive effects. Learning motivation had a path coefficient of 0.210 (SE = 0.061, CR = 3.458) and reflective behavior had a path coefficient of 0.400 (SE = 0.062, CR = 6.479), both of which were highly significant. In summary, timely corrective feedback and reflection task significantly stimulated both reflective behavior and intrinsic learning motivation. Recognition accuracy mainly affected learning motivation, while system usage frequency moderately promoted reflective behavior but did not significantly influence learning motivation. At the same time, reflective behavior and learning motivation further contributed directly to the improvement of students’ oral proficiency.

To test the moderating effects of language proficiency hypothesized in H11a and H11b, we employed multi-group structural equation modeling (multi-group SEM). Language proficiency was measured as a categorical variable with three levels: beginner (*n* = 183), intermediate (*n* = 95), and advanced (*n* = 47), based on participants’ self-assessments adapted from Marian et al. (2007). Following established procedures for testing moderation with categorical variables in SEM (Byrne, 2016; Kline, 2023), we conducted the analysis in two steps. First, we established a baseline model in which all path coefficients were freely estimated across the three proficiency groups. This model provided the baseline chi-square value (*χ*^2^) and degrees of freedom (df) against which constrained models would be compared.

Second, the study tested for moderating effects by comparing the baseline model with a series of constrained models in which specific paths of interest were forced to be equal across groups. Specifically, to test H11a (moderating effect of language proficiency on the relationship between reflective behavior and oral proficiency improvement), we constrained the path from reflective behavior to oral proficiency improvement to be equal across the three groups and compared the model fit with the baseline model. A significant chi-square difference (Δ*χ*^2^) between the constrained and baseline models would indicate that the path coefficient differs significantly across proficiency levels, thus supporting the moderating hypothesis (Hair et al., 2019). The same procedure was applied to test H11b (moderating effect of language proficiency on the relationship between learning motivation and oral proficiency improvement), by constraining the path from learning motivation to oral proficiency improvement to be equal across groups. If a significant chi-square difference was detected, we further examined the pattern of moderation by inspecting the unstandardized path coefficients for each group and conducting pairwise comparisons to identify which specific groups differed significantly. This multi-group approach is widely recommended for testing moderation with categorical variables in SEM (Wang and Wang, 2020) and ensures that the analysis appropriately reflects the categorical nature of the language proficiency variable.

### 4.6 Moderating effects analysis

To test the moderating effects of language proficiency hypothesized in H11a and H11b, we employed multi-group structural equation modeling (multi-group SEM). Language proficiency was measured as a categorical variable with three levels: beginner (*n* = 183), intermediate (*n* = 95), and advanced (*n* = 47), based on participants’ self-assessments adapted from Marian et al. (2007) and aligned with CEFR criteria as described in Section 4.1.

Following established procedures for testing moderation with categorical variables in SEM (Byrne, 2016; Kline, 2023), the analysis was conducted in two steps. First, a baseline model was established in which all path coefficients were freely estimated across the three proficiency groups. This baseline model yielded a chi-square value (*χ*^2^ = 892.47, df = 1,476) against which constrained models would be compared. Second, moderating effects were tested by comparing the baseline model with a series of constrained models in which specific paths of interest were forced to be equal across groups. Specifically, to test H11a (moderating effect of language proficiency on the relationship between reflective behavior and oral proficiency improvement), the path from reflective behavior to oral proficiency improvement was constrained to be equal across the three groups, and the model fit was compared with the baseline model.

A significant chi-square difference (Δ*χ*^2^) between the constrained and baseline models would indicate that the path coefficient differs significantly across proficiency levels, thus supporting the moderating hypothesis (Hair et al., 2019). The same procedure was applied to test H11b (moderating effect of language proficiency on the relationship between learning motivation and oral proficiency improvement), by constraining the path from learning motivation to oral proficiency improvement to be equal across groups. If a significant chi-square difference was detected, the pattern of moderation was further examined by inspecting the unstandardized path coefficients for each group and conducting pairwise comparisons to identify which specific groups differed significantly.

#### 4.6.1 Moderating role of students’ language proficiency on the relationship between reflective behavior and oral proficiency improvement

This study examines the moderating role of language proficiency in the effect of reflective behavior on oral proficiency improvement. Table 8 shows that the model’s constant is estimated at 3.6725. Reflective behavior has a significant direct effect on oral proficiency improvement, with a coefficient of 0.2529 (SE = 0.0487, CR = 5.1892, *p* < 0.001). language proficiency also shows a significant direct effect, with a coefficient of 0.5081 (SE = 0.0570, CR = 8.9173, *p* < 0.001), indicating that both factors positively contribute to oral improvement. Crucially, the interaction term between reflective behavior and language proficiency is significant, with a coefficient of 0.1959 (SE = 0.0481, CR = 4.0706, *p* < 0.001). This finding indicates that as language proficiency improves, the effect of reflective behavior on oral proficiency improvement is significantly enhanced.

**Table 8:** Results of students’ language proficiency adjusted to reflective behavior and oral proficiency improvement.

Experimental result				
Model path	Coefficient (Estimate)	Standard error (S.E.)	Critical ratio (C.R.)	*p* value
Constant	3.6725	0.0482	76.1916	0.0000
Reflective behavior	0.2529	0.0487	5.1892	0.0000
Language proficiency	0.5081	0.0570	8.9173	0.0000
Reflective behavior × Language proficiency	0.1959	0.0481	4.0706	0.0001

Conditional effects of regulatory effects						
Language proficiency	(Effect)	Standard error (S.E.)	Critical ratio (t)	*p* value	Lower limit (LLCI)	Upper limit (ULCI)
−0.8721	0.0820	0.0616	1.3309	0.1842	−0.0392	0.2032
0.0000	0.2529	0.0487	5.1892	0.0000	0.1570	0.3488
0.8721	0.4238	0.0669	6.3325	0.0000	0.2921	0.5554

Further conditional effects analysis reveals that when language proficiency is low, the effect of reflective behavior on oral improvement is weak and not significant (conditional effect estimate = −0.8721, SE = 0.0820, CR = 1.3309, *p* = 0.1842, 95% CI [−0.0392, 0.2032]). At the average level, the positive effect is significantly enhanced (conditional effect estimate = 0.0000, SE = 0.2529, CR = 5.1892, *p* < 0.001, 95% CI [0.1570, 0.3488]). When language proficiency is high, the promotive effect of reflective behavior on oral proficiency improvement becomes even more apparent (conditional effect estimate = 0.8721, SE = 0.4238, CR = 6.3325, *p* < 0.001, 95% CI [0.2921, 0.5554]). These results clearly demonstrate that in ASR-assisted oral instruction, a solid language proficiency can effectively enhance the positive effect of reflective behavior on oral proficiency, thereby better transforming external feedback into actual language improvement.

#### 4.6.2 Moderating role of language proficiency on the relationship between learning motivation and oral proficiency improvement

This study further explores the moderating effect of language proficiency on the impact of learning motivation on oral proficiency improvement. Table 9 shows that the model’s constant is 3.7220. Learning Motivation has a significant direct positive effect on oral proficiency improvement, with a path coefficient of 0.1744 (SE = 0.0476, CR = 3.6672, *p* = 0.0003). At the same time, language proficiency also exhibits a significant direct positive effect, with a path coefficient of 0.5666 (SE = 0.0548, CR = 10.3319, *p* < 0.001). Crucially, the interaction term between learning motivation and language proficiency has a coefficient of 0.1200 (SE = 0.0515, CR = 2.3294, *p* = 0.0205). This indicates that as the level of language proficiency increases, the effect of learning motivation on oral proficiency improvement becomes markedly stronger. Further conditional effects analysis reveals that when language proficiency is low, the effect of learning motivation on oral improvement is weak and not significant (conditional effect estimate = 0.0698, SE = 0.0664, CR = 1.0516, *p* = 0.2938, 95% CI [−0.0608, 0.2003]). At an average level, the positive effect is significantly enhanced (conditional effect estimate = 0.1744, SE = 0.0476, CR = 3.6672, *p* = 0.0003, 95% CI [0.0808, 0.2680]). When language proficiency is high, the promotive effect of learning motivation is further increased (conditional effect estimate = 0.2790, SE = 0.0645, CR = 4.3288, *p* < 0.001, 95% CI [0.1522, 0.4058]). These results fully demonstrate that in ASR-assisted oral instruction, a strong language proficiency can significantly enhance the conversion of learning motivation into improvements in oral proficiency.

**Table 9:** Results of language proficiency adjusted to learning motivation and oral proficiency improvement.

Experimental result				
Model path	Coefficient (Estimate)	Standard error (S.E.)	Critical ratio (C.R.)	*p* value
Constant	3.7220	0.0472	78.7789	0.0000
Learning motivation	0.1744	0.0476	3.6672	0.0003
Language proficiency	0.5666	0.0548	10.3319	0.0000
Learning motivation × Language proficiency	0.1200	0.0515	2.3294	0.0200

Conditional effects of regulatory effects						
Language proficiency	(Effect)	Standard error (S.E.)	Critical ratio (*t*)	*p* value	Lower limit (LLCI)	Upper limit (ULCI)
−0.8721	0.0698	0.0664	1.0516	0.2938	−0.0608	0.2003
0.0000	0.1744	0.0476	3.6672	0.0003	0.0808	0.2680
0.8721	0.2790	0.0645	4.3288	0.0000	0.1522	0.4058

### 4.7 Discussion

**Figure 3:**
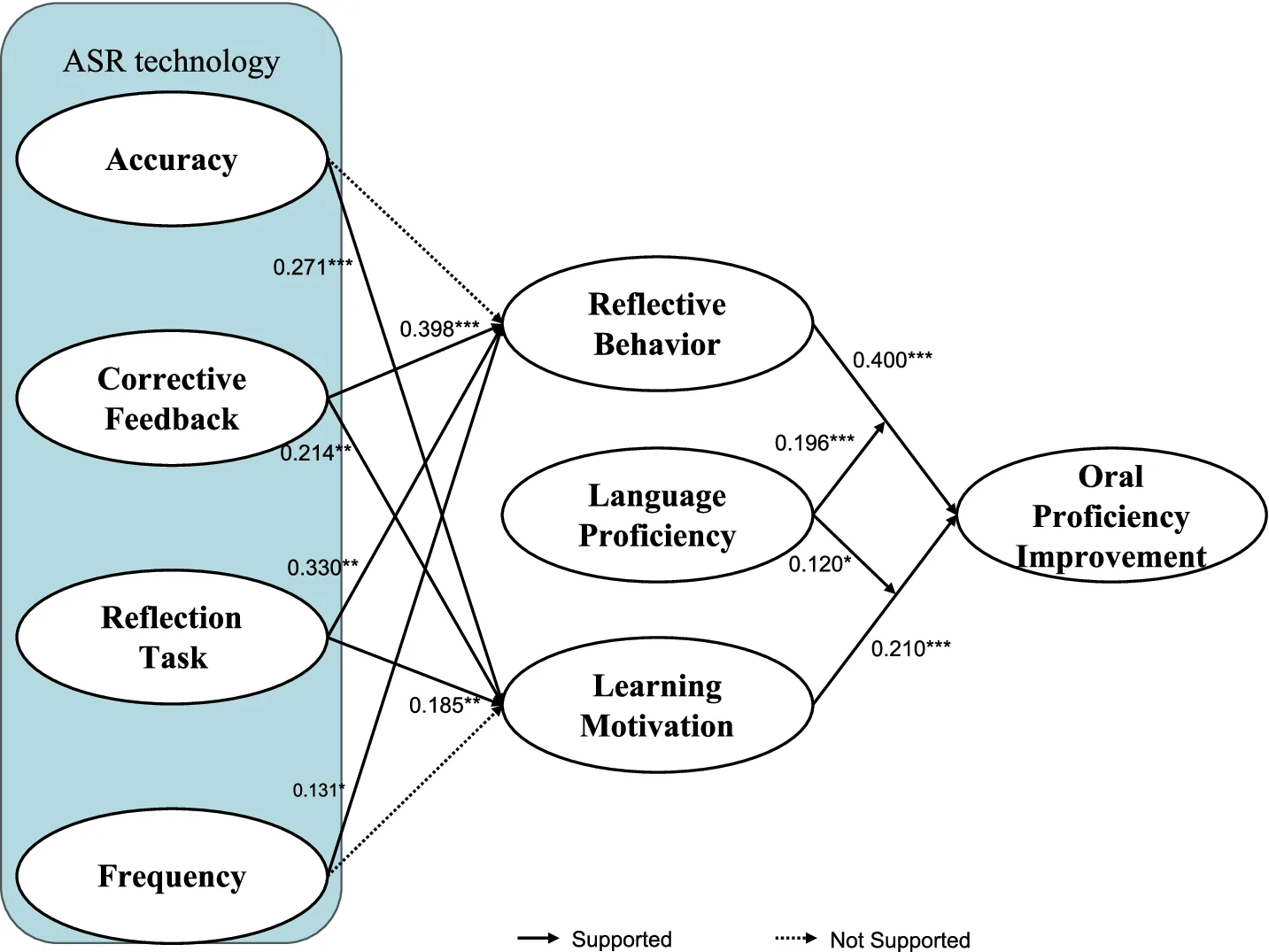
Final structural model with standardized path coefficients. *p < 0.05, **p < 0.01, ***p < 0.001.

This study constructed a multidimensional integrated theoretical model to examine the application of ASR technology in college oral instruction, exploring the complex interactions between technological interventions and learners’ internal cognitive processes, and how external feedback is transformed into language skill improvement through self-reflection and self-regulation. Overall, the findings largely support the proposed theoretical framework, while also revealing several nuanced insights that contribute to a deeper understanding of ASR-mediated language learning (see Figure 3).

The results revealed differential effects of the four ASR technological features on learners’ reflective behavior and learning motivation. Specifically, H1 predicted a positive relationship between recognition accuracy and reflective behavior, but this hypothesis was not supported. This unexpected finding suggests that the system’s speech recognition capability alone may not directly trigger deep reflection in students. A possible explanation is that accurate error detection, while necessary, is insufficient to stimulate metacognitive engagement unless accompanied by pedagogical scaffolding that prompts learners to act on the feedback. Learners may simply note the error flagged by the ASR system without engaging in deeper analysis of its causes or strategies for improvement (Li, 2010). This finding implies that instructional design should focus on the depth of feedback and the specificity of reflection tasks rather than solely on technical accuracy.

In contrast, H3 and H5 were supported, showing that corrective feedback and reflection tasks significantly enhanced reflective behavior. These results confirm that timely, clear corrective feedback stimulates learners’ self-examination and strategy adjustment, while structured reflection tasks such as listening to recordings and maintaining reflective journals provide essential frameworks for self-assessment (Farrell, 2018; Richards and Lockhart, 1994). For learning motivation, H2, H4, and H6 were supported, indicating that accuracy, corrective feedback, and reflection tasks all positively predicted intrinsic motivation. Notably, while accuracy did not directly drive reflection, it did enhance motivation. This suggests that learners’ confidence in the feedback they receive, along with their trust that the system is correctly identifying their errors, may be a prerequisite for sustained engagement, even if it does not automatically lead to deeper cognitive processing (Liakin et al., 2017).

H7 was supported (*β* = 0.131, *p* = 0.021), showing that usage frequency had a modest but significant effect on reflective behavior, confirming that frequent system use provides abundant practice opportunities and moderately stimulates reflective engagement. However, H8 was not supported (*β* = 0.048, *p* = 0.402), indicating that frequency did not significantly predict learning motivation. This null finding is particularly noteworthy, as it challenges the assumption that mere exposure or practice frequency automatically enhances motivation. Rather, it suggests that the quality of interaction, the depth of feedback, and the significance of reflective tasks are more important than the quantity of system interaction (Golonka et al., 2014). Turning to the effects of metacognitive strategies on learning outcomes, H9 and H10 were both supported. Reflective behavior and learning motivation both significantly predicted oral proficiency improvement. The stronger effect of reflective behavior compared to learning motivation suggests that in ASR-mediated learning environments, the cognitive process of reflection may be more directly linked to skill development than affective factors alone. However, the significant contribution of both variables confirms that effective learning requires the integration of cognitive and affective processes (Oxford, 2017).

A key contribution of this study is the empirical demonstration of language proficiency as a critical moderator of the relationships between metacognitive strategies and oral proficiency outcomes, supporting H11a and H11b. For the relationship Reflective Behavior and Oral Proficiency Improvement (H11a), the significant interaction term indicates that the positive effect of reflection on speaking gains is significantly enhanced for students with stronger language proficiency. Conditional effects analysis revealed that at low levels of proficiency, the effect of Reflective Behavior was weak and non-significant, while at high proficiency levels, the effect was substantially stronger. This pattern suggests that less proficient learners may lack the foundational knowledge necessary to benefit from reflective practice, echoing the challenges identified in the literature on feedback processing (Flege, 1995; Nation, 2013).

Similarly, for the relationship between learning motivation and oral proficiency improvement (H11b), the significant interaction term demonstrates that motivation translates more effectively into improvement for students with stronger language foundations. The effect was non-significant at low proficiency levels but significant and increasingly stronger at average and high proficiency levels. These moderating effects align with theoretical perspectives on the role of prior knowledge in feedback processing. Students with stronger language proficiency possess more developed phonological categories, larger lexical repertoires, and more automatized grammatical processing, enabling them to quickly and accurately interpret corrective feedback and translate it into improvement (Kormos, 2014; Segalowitz, 2010).

The findings also have important implications for addressing the potential psychological drawbacks of ASR use discussed earlier in the literature review. As noted by Saito et al. (2020), Suzuki (2025), and Leis (2025), ASR’s precise feedback can lead to decreased self-confidence when learners realize their actual pronunciation falls short of their self-perceptions - a manifestation of the Dunning-Kruger effect. The present results suggest that this risk may be particularly acute for lower-proficiency learners, who receive frequent error notifications and may become discouraged. This underscores the need for instructional designs that balance accurate feedback with supportive scaffolding, such as positive framing of errors as learning opportunities and gradual increases in feedback complexity as learners progress.

The findings also have practical implications for adaptive instructional design. The non-significant effects at low proficiency levels suggest that one-size-fits-all approaches to ASR implementation may inadvertently widen achievement gaps. Students with weaker language foundations may need additional support structures, such as simplified feedback, more explicit guidance on reflection strategies, or supplementary instruction to benefit equally from ASR technology (Ranalli, 2021; Stevenson and Phakiti, 2014). In summary, this study advances theoretical understanding of ASR-mediated language learning by specifying the differential effects of technological features, the mediating role of metacognitive strategies, and the critical moderating function of language proficiency.

## 5 Implications

### 5.1 Theoretical implications

This study makes several theoretical contributions to the fields of technology-enhanced language learning and second language acquisition. First, by integrating ASR technological features, metacognitive strategies, and individual differences into a unified model, we provide a more comprehensive understanding of how technology-mediated feedback translates into learning outcomes. The differential effects of the four ASR features challenge simplistic views of technology effectiveness and highlight the need for nuanced theoretical frameworks that distinguish between technical capabilities and pedagogical design. Second, the findings extend self-regulated learning theory (Zimmerman, 2000; Oxford, 2017) to the context of ASR-mediated speaking instruction, demonstrating that reflective behavior and motivation serve as crucial mediating mechanisms between technological input and learning outcomes. The strong effect of reflective behavior confirms that metacognitive engagement is essential for transforming external feedback into internalized knowledge. Third, the moderating effects of language proficiency contribute to individual differences research by specifying conditions under which metacognitive strategies are effective. These findings support DeKeyser's (2012) emphasis on the interaction between learner characteristics and instructional interventions and suggest that theories of feedback effectiveness must account for learners’ developmental readiness to process and utilize feedback. Specifically, the findings indicate that reflective practice and motivation are not universally effective strategies; their benefits depend on learners having sufficient linguistic foundation to act upon them.

### 5.2 Practical implications

The findings provide concrete guidance and operational recommendations for actual teaching practice. For educators, effective ASR integration requires more than simply providing access to technology. First, prioritize feedback quality over practice frequency. The non-significant effect of frequency on motivation indicates that mere exposure without pedagogical value does not enhance motivation. Teachers should select ASR systems that provide pedagogically sound explanations rather than simple error detection. An example of a pedagogically sound explanation: When a learner mispronounces the English /θ/ sound (as in “think”) as /s/ (as in “sink”), rather than simply flagging “pronunciation error,” the ASR system should provide targeted articulatory guidance: “Your tongue should be placed between your upper and lower teeth to produce the /θ/ sound. Listen to the difference: /θɪŋk/ vs. /sɪŋk/. Now try again, placing your tongue between your teeth and blowing air gently.” This type of explanation connects the error to a specific articulatory action and provides a clear path for correction.

Second, scaffold reflection through structured tasks. The significant effects of reflection tasks on both reflective behavior and motivation confirm their importance. Teachers should integrate guided reflection activities such as listening to recordings, maintaining reflective journals, and setting specific pronunciation goals. To facilitate structured reflection, teachers may incorporate prompts such as the following into ASR-based practice activities. A self-observation prompt asks learners: “Listen to your recorded sentence and compare it with the native speaker model. What is ONE specific difference you notice in pronunciation, intonation, or rhythm?” A self-judgment prompt asks: “How successfully did you produce the target sound in your second attempt compared to your first? What evidence supports your rating?” A self-reaction prompt asks: “What is ONE concrete strategy you will use to improve the most challenging sound in this word before your next practice session?” Finally, a goal-setting prompt asks: “Based on today’s feedback, what is a realistic pronunciation goal for your next 10-minute ASR practice session?”

Third, differentiate instruction based on proficiency levels. The moderating effects reveal that less proficient learners benefit less from reflective practice and motivation alone, suggesting they need simplified feedback and explicit strategy guidance, while advanced learners benefit from more complex reflection tasks. For beginners, teachers should provide additional scaffolding such as pre-teaching articulatory concepts, modeling reflection processes, and celebrating small improvements to maintain motivation. For advanced learners, teachers can assign more open-ended reflection tasks that require critical analysis of subtle pronunciation features.

For technology developers, the findings highlight three design priorities. First, enhance feedback quality. Rather than simple binary (correct/incorrect) indicators, systems should provide pedagogically sound explanations that include articulatory instructions, contrastive examples, and visual feedback (e.g., waveform displays, tongue position diagrams). Second, integrate structured reflection tools directly into system interfaces, including one-click recording playback, progress tracking dashboards, and guided self-assessment prompts such as those listed above. Third, develop adaptive systems that personalize feedback complexity based on learners’ proficiency levels, ensuring that beginners receive simplified, supportive feedback while advanced users engage in deeper metacognitive challenges.

### 5.3 Methodological implications

This study offers several methodological contributions for future research on technology-mediated language learning. First, the use of multi-group structural equation modeling (SEM) to test the moderating effects of a categorical variable (language proficiency with three levels) provides a replicable analytical framework. As described in Section 4.6, it is recommended to use a two-step approach - establishing a baseline model with a freely estimated path and then conducting a chi square test on the constrained model - to test the moderating effect of categorical variables (Byrne, 2016; Kline, 2023). Second, the comprehensive validation process for the measurement instrument (expert review, pilot testing, EFA, and CFA) described in Section 4.1 serves as a model for developing psychometrically sound instruments in computer-assisted language learning research. Future studies investigating similar constructs are encouraged to adopt or adapt these validated scales. Third, the finding that certain paths (e.g., accuracy to reflective behavior) were non-significant while others (e.g., corrective feedback to reflective behavior) were significant underscores the importance of disaggregating technology features rather than treating ASR as a monolithic intervention. Future research should continue to examine the unique contributions of specific design elements rather than comparing “ASR use” versus “non-use” globally. Finally, the differential conditional effects across proficiency levels highlight the importance of reporting simple slopes and interaction plots when presenting moderation results, as done in Tables 8, 9. This practice allows readers to assess not only whether moderation exists but also at which levels of the moderator the effects are significant.

## 6 Limitations and future research

Several important caveats of this research deserve attention, and addressing these limitations offers productive directions for future investigation.

First, the cross-sectional design precludes causal inferences about the relationships between ASR features, metacognitive strategies, and learning outcomes. Future research should employ experimental or longitudinal designs to track students’ development across multiple time points and to investigate the cumulative effects of ASR-mediated practice. Randomized controlled trials comparing ASR-supported instruction with traditional instruction would provide stronger evidence for causal claims.

Second, the sample was drawn from a single university in China, which may limit the generalizability of findings to other cultural and educational contexts. Chinese learners may have specific characteristics, such as pronunciation challenges, learning traditions, or technology familiarity that influence how they interact with ASR technology. Cross-cultural comparative studies would be valuable for examining whether the observed relationships hold across diverse learner populations and educational settings. Replicating this study with learners from different language backgrounds (e.g., Spanish, Arabic, Japanese speakers) and different instructional contexts (e.g., high school, adult education, self-directed learning) would enhance the external validity of the findings.

Third, the study did not control for teacher variables or classroom environments, which may influence how students engage with ASR technology. Factors such as teacher guidance, peer collaboration, and curricular integration may moderate the effectiveness of ASR-mediated learning. Additionally, the high Cronbach’s alpha value (0.915) observed for the overall scale, while indicating strong internal consistency, may also suggest some degree of item redundancy. This does not invalidate the reliability of the instrument, but future research may consider refining the scale by removing potentially redundant items to improve efficiency and parsimony.

Future research should examine these contextual factors and investigate how ASR technology can be optimally integrated into broader instructional frameworks. Qualitative studies, such as classroom observations and learner interviews, would complement the quantitative findings by providing insights into how and why learners engage with ASR features in authentic classroom settings. Despite these limitations, this study provides robust evidence for the differential effects of ASR features on metacognitive strategies and the critical moderating role of language proficiency. These findings advance theoretical understanding of technology-mediated language learning and offer practical guidance for optimizing ASR-based instruction to meet the needs of diverse learners.

## 7 Conclusion

This study examined how ASR technology features interact with metacognitive strategies and learner differences to influence oral English improvement. The findings reveal a nuanced picture of ASR-mediated learning. The findings reveal that corrective feedback and reflection tasks significantly enhance students’ reflective behavior and motivation, while usage frequency alone does not boost motivation. Moreover, recognition accuracy influences motivation but not reflection, suggesting that technical precision alone is insufficient to promote deeper cognitive engagement. Both reflective behavior and motivation positively predict oral proficiency gains. Importantly, language proficiency moderates these effects: stronger learners internalize feedback more effectively, while weaker learners may need additional support. This finding underscores the importance of adaptive instructional design that responds to learners’ developmental readiness. For practice, educators should prioritize feedback quality over quantity, scaffold reflection through structured tasks, and differentiate instruction by proficiency level. Developers should design systems with pedagogically sound feedback (such as articulatory explanations rather than simple error flags) and adaptive features that personalize feedback complexity. Institutions should invest in quality technology and ensure equitable support for all learners, particularly those with lower proficiency who may require additional scaffolding. In sum, ASR technology holds significant potential for enhancing oral English instruction, but its effectiveness hinges on thoughtful pedagogical integration. By combining high-quality feedback, structured reflection, and differentiated support, educators and developers can create learning environments that help all students, regardless of proficiency level, to develop the speaking skills needed for academic and professional success in an interconnected world.

## Data Availability

The raw data supporting the conclusions of this article will be made available by the authors, without undue reservation.
